# Electroacupuncture Relieves Neuropathic Pain by Suppressing ACC Pyramidal Activity via mGluR5 Inhibition in CCI Mice

**DOI:** 10.1155/np/9427819

**Published:** 2026-05-13

**Authors:** Xiuxiu Zhuang, Ledan Huang, Fubei Nan, Shuangdong Chen, Junlu Wang, Yunchang Mo, Anqi Zhang

**Affiliations:** ^1^ Department of Anesthesiology, The First Affiliated Hospital of Wenzhou Medical University, Wenzhou, 325000, Zhejiang, China, wzhospital.cn

**Keywords:** ACC, CCI, chemogenetic, electroacupuncture, mGluR5, neuropathic pain, optogenetics

## Abstract

**Background:**

Electroacupuncture (EA) is widely used for analgesia, but its central mechanisms remain unclear. We investigated whether EA alleviates neuropathic pain by suppressing metabotropic glutamate receptor 5 (mGluR5) signaling in the anterior cingulate cortex (ACC).

**Methods:**

In naïve mice, we manipulated ACC pyramidal neurons using adeno‐associated viral (AAV) vectors encoding calcium/calmodulin‐dependent protein kinase II (CaMKII)‐driven opsins—channelrhodopsin‐2 (ChR2) or halorhodopsin (NpHR3.0)—followed by blue‐ or yellow‐light stimulation to assess behavioral responses. In a chronic constriction injury (CCI) model, mice received EA and were evaluated for mechanical and thermal withdrawal thresholds. Western blotting (WB) and immunofluorescence (IF) quantified ACC mGluR5 expression. Then CaMKII‐targeted adeno‐associated viruses expressing chemogenetic receptors, such as hM3Dq or hM4Di, were injected into the ACC. Two weeks later, CCI was induced, and mice received either EA or intraperitoneal clozapine‐N‐oxide (CNO) while pain behaviors were monitored. Finally, proteomic profiling of ACC tissue compared CCI and EA groups.

**Results:**

Optogenetic activation of ACC pyramidal neurons in naïve mice reduced both mechanical and thermal withdrawal thresholds, indicating a pronociceptive effect, whereas optogenetic inhibition increased thresholds. In CCI mice, EA significantly attenuated hypersensitivity and downregulated ACC mGluR5 protein levels by WB and IF. Chemogenetic inhibition of ACC pyramidal neurons similarly elevated thresholds in CCI mice, imitating EA. Notably, combining chemogenetic inhibition with EA produced no additional improvement, suggesting convergence on a common ACC mGluR5‐dependent pathway.

**Conclusions:**

EA relieves neuropathic pain in mice, at least in part, by suppressing ACC pyramidal neuron activity via inhibition of mGluR5 signaling.

## 1. Introduction

Neuropathic pain is associated with persistent alterations in cortical circuits involved in pain perception and emotional processing [1]. Among these regions, the anterior cingulate cortex (ACC) plays a crucial role in integrating the sensory and affective dimensions of pain. Increasing evidence indicates that peripheral nerve injury induces long‐lasting synaptic plasticity within ACC circuits, including enhanced excitatory synaptic transmission and long‐term potentiation (LTP)‐like mechanisms in pyramidal neurons [2, 3]. These maladaptive plasticity changes contribute to cortical hyperexcitability and are considered key mechanisms underlying the persistence of chronic pain.

The ACC is a critical hub for integrating sensory and emotional components of pain. It is consistently activated in both acute and chronic pain conditions, with pyramidal neurons demonstrating increased excitability and synaptic potentiation following peripheral injury [4–6]. Optical genetics technology has recently revealed that activation of ACC pyramidal neurons can induce hyperalgesia in naïve mice, while inhibition of these neurons can relieve chronic pain in mice [7]. Manipulating ACC pyramidal neurons through optogenetics or pharmacological inhibition has been shown to modulate both nociceptive and affective dimensions of chronic pain [8, 9]. In chronic neuropathic pain states, pyramidal neuron hyperexcitability and maladaptive plasticity in the ACC have been repeatedly implicated in both nociceptive gain and negative affect [10].

Glutamatergic neurotransmission is a key component of pain processing and neuroplasticity. Activation of glutamate receptors, including NMDA receptors and metabotropic glutamate receptors, regulates excitatory synaptic transmission and neuronal excitability in cortical circuits. Among them, metabotropic glutamate receptor 5 (mGluR5) has been implicated in the modulation of synaptic plasticity and central sensitization during chronic pain [11]. Increased mGluR5 signaling in cortical circuits can potentiate glutamatergic transmission, reduce nociceptive thresholds, and reinforce aversive pain‐related learning [12]. Astragalin was shown to relieve inflammatory pain and negative emotions by inhibiting mGluR5 signaling in the ACC and hypothalamus [13]. In addition to neuronal expression, transient re‐expression of mGluR5 in astrocytes during early stages of chronic pain has been reported to promote excitatory synaptogenesis and mechanical hypersensitivity, suggesting that neuron‐glia interactions may further amplify ACC hyperexcitability [14]. Transient re‐expression of mGluR5 in cortical astrocytes during the early stages of chronic pain was found to promote excitatory synaptogenesis and mechanical nociceptive sensitization [15]. Increased mGluR5 signaling in cortical circuits can potentiate glutamatergic drive, lower withdrawal thresholds, and reinforce aversive learning. Early‐phase glial responses (for example, transient astrocytic mGluR5 re‐expression) further suggest a neuron‐glia‐synapse axis that fuels ACC hyperexcitability. These observations make mGluR5 a biologically plausible target for circuit‐level analgesia.

Electroacupuncture (EA), a technique that combines traditional acupuncture with electrical stimulation, is a well‐established intervention in complementary and alternative medicine. It offers precise control over stimulation parameters and has shown robust efficacy in pain relief, primarily by promoting the release of endogenous opioid peptides and modulating neural activity in specific brain regions [16, 17]. EA has been shown to attenuate glutamate‐induced excitotoxicity by downregulating pro‐apoptotic markers, such as caspase‐3, Bax, and pJNK [18]. This mechanism may also be involved in its therapeutic effects in neuropsychiatric disorders, such as post‐traumatic stress disorder (PTSD), where glutamatergic dysfunction and hippocampal neuronal damage contribute to re‐experiencing and anxiety symptoms [19, 20]. EA’s modulation of mGluR5 signaling has been linked to the suppression of central sensitization and migraine‐associated allodynia, potentially via mTOR pathway regulation and autophagy activation [21, 22]. These findings suggest that regulation of glutamatergic signaling may represent an important mechanism underlying the analgesic effects of EA.

In parallel, accumulating studies indicate that EA alters neural activity within the limbic‐paralimbic‐neocortical network, including the ACC. fMRI and animal studies indicate that acupuncture, especially at points like ST36, can modulate the cAMP/PKA/CREB signaling pathway in the ACC and reduce pain‐related memory and behavior [23–25]. In inflammatory and neuropathic pain models, EA‐induced analgesia has been attenuated by direct ACC manipulation, further implicating this region in its effects [26]. Prior work shows that EA can modulate cortical and subcortical nodes involved in pain regulation and, in some contexts, downshift glutamatergic signaling [27]. Despite these advances, two key questions remain unresolved. First, direct causal evidence that EA alleviates neuropathic pain by suppressing the activity of ACC pyramidal neurons remains limited. Second, it remains unclear whether EA‐induced analgesia involves modulation of mGluR5‐dependent glutamatergic signaling within ACC circuits.

To address these issues, we combined EA with chemogenetic manipulation of ACC pyramidal neurons in a mouse model of neuropathic pain. Using designer receptors exclusively activated by designer drugs (DREADDs), we selectively activated or inhibited ACC excitatory neurons and examined the resulting effects on pain behaviors and mGluR5 expression. By integrating behavioral assays with molecular and proteomic analyses, we aimed to determine whether EA alleviates neuropathic hypersensitivity by suppressing ACC pyramidal neuron excitability and modulating mGluR5‐mediated glutamatergic signaling. We aimed to determine whether EA attenuates neuropathic hypersensitivity by suppressing ACC pyramidal excitability via mGluR5 inhibition.

## 2. Material and Methods

### 2.1. Experimental Animals and Group

Adult male C57BL/6 mice (6–8 weeks old) were used. All procedures in this study were approved by the Laboratory Animal Ethics Committee of the First Affiliated Hospital of Wenzhou Medical University to ensure minimal animal use and discomfort (Number: WYYY‐AEC‐2023‐010). Male C57BL/6 mice were purchased from Zhejiang Vital River Laboratory Animal Technology Co., Ltd. Mice were kept on a 12 h light/dark cycle, with food and water provided ad libitum. Animals were acclimated to the behavioral testing room for 7 days, and then randomly divided into different treatment groups (see Table 1). The meaning of the group is as follows:

**Table 1:** Grouping.

Group	Virus	Light	Intraperitoneal injection (i.p) CNO or normal saline (NS)	Electroacupuncture (EA)
ChR2‐on	AAV2/9‐CamkII‐ChR2‐mcherry	473 nm	–	–
ChR2‐off	AAV2/9‐CamkII‐ChR2‐mcherry	–	–	–
NpHR‐on	AAV2/9‐CamkII‐eNpHR3.0‐GFP	589 nm	–	–
NpHR‐off	AAV2/9‐CamkII‐eNpHR3.0‐GFP	–	–	–
EA + 3Dq + NS	AAV2/9‐CamkII‐hM3Dq‐mcherry	–	NS	EA
3Dq + CNO	AAV2/9‐CamkII‐hM3Dq‐mcherry	–	CNO	–
mcherry + CNO	AAV2/9‐CamkII‐mcherry	–	CNO	–
EA + 4Di + CNO	AAV2/9‐CamkII‐hM4Di‐GFP	–	CNO	EA
EA + 4Di + NS	AAV2/9‐CamkII‐hM4Di‐GFP	–	NS	EA
4Di + CNO	AAV2/9‐CamkII‐hM4Di‐GFP	–	CNO	–
4Di + NS	AAV2/9‐CamkII‐hM4Di‐GFP	–	NS	–
GFP + CNO	AAV2/9‐CamkII‐GFP	–	CNO	–

The channelrhodopsin‐2 (ChR2)‐on group was injected with adeno‐associated viral (AAV)2/9‐calcium/calmodulin‐dependent protein kinase II (CaMKII)α‐ChR2‐mCherry, and cannulas were implanted into the ipsilateral (ips) ACC and stimulated by blue light (on), while the contralateral (con) plantar was measured. Other group names are similar.

The 3Dq + clozapine‐N‐oxide (CNO)‐ips group was injected with AAV2/9‐CamkII‐hM3Dq‐mcherry into the ips ACC and stimulated by CNO; after 45 min, the ips plantar was measured. Other group names are similar.

The EA + 3Dq + CNO‐con group was injected with AAV2/9‐CamkII‐hM3Dq‐mcherry into the ips ACC and stimulated by CNO. After 15 min, EA stimulation was administered for 30 min, the needle was removed, and then the con plantar was measured. Other group names are similar.

### 2.2. Chronic Constriction Injury (CCI)

**Figure 1:**
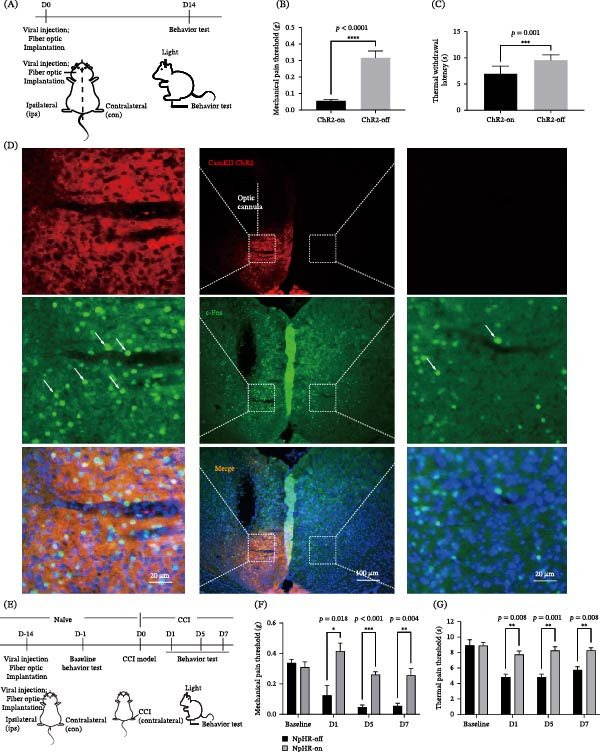
Optogenetic activation or inhibition of ACC pyramidal neurons bidirectionally regulates nociceptive thresholds. (A) Experimental schematic activation of ACC pyramidal neurons. Viral injection and fiber implantation were performed in the ipsilateral (ips) of ACC, while CCI surgery was carried out on the contralateral (con) sciatic nerve. Here, ips indicates the injection/fiber side, and con indicates the side receiving nerve injury. (B and C) The paw of mechanical pain thresholds and thermal withdrawal threshold values when photoactivation of ACC excitatory neurons (ChR2‐on) in naïve mice compared with ChR2‐off controls (Student’s *t* test, *n* = 8,  ^∗∗∗^ *p*  < 0.001,  ^∗∗∗∗^ *p*  < 0.0001). (D) c‐Fos immunostaining confirmed robust neuronal activation in the ACC following blue light stimulation. (E) Experimental schematic inhibition of ACC pyramidal neurons. (F and G) The mechanical pain threshold and thermal pain threshold values when yellow light‐mediated (589 nm) inhibition of ACC excitatory neurons in CCI mice (*n* = 10, repeated measures ANOVA with a Bonferroni post hoc test,  ^∗^ *p*  < 0.05,  ^∗∗^ *p*  < 0.01,  ^∗∗∗^ *p*  < 0.001).

CCI was performed as described previously [28]. Mice were deeply anesthetized with 1.25%–1.5% isoflurane, and the con sciatic nerve was exposed at the mid‐thigh level (Figure 1A). After the removal of adherent tissue, three loose ligatures with chromic gut 6/0 were tied 1 mm apart around the nerve. Muscle and skin were closed with sutures (Silkam 6/0 and Safil 4/0, respectively; Ethicon).

### 2.3. Stereotaxic Cannula Implantation and Intracranial Viral Injections

As previously described, mice were anesthetized with isoflurane (1.25%–1.5%). For the ips ACC (Figure 1A), mice were unilaterally injected with 0.5 µl of AAV2/9‐CamkII‐ChR2‐mcherry (1.1 × 10^12^ vg/mL, Hanbio Biotechnolog, China), which is sensitive to blue light (473 nm) to excite neurons, and AAV2/9‐CamkII‐eNpHR3.0‐GFP (1.5 × 10^12^ vg/mL, Hanbio Biotechnolog, China), which is sensitive to yellow light (589 nm) to inhibit neurons, as well as AAV2/9‐CamkII‐hM3Dq‐mcherry (1.0 × 10^12^ vg/mL, Hanbio Biotechnolog, China), AAV2/9‐CamkII‐hM4Di‐GFP (1.2 × 10^12^ vg/mL, Hanbio Biotechnolog, China), AAV2/9‐CamkII‐mcherry, and AAV2/9‐CamkII‐GFP, at a rate of 0.5 µl/10 min, using a 33‐gauge, 10 µl Hamilton syringe anteroposteriorly (AP) + 0.98 mm, mediolaterally (ML) + 0.32 mm, and dorsoventrally (DV)—1.6 mm, according to the Mouse Brain in Stereotaxic Coordinates, second edition atlas, by George Paxinos. The microinjection was held in place for 10 min to allow for the diffusion of virus particles away from the injection site and to minimize the spread of viral particles along the injection track. Mice were then unilaterally implanted with a guide cannula (PlasticsOne) in the ips ACC: AP + 0.98 mm, ML + 0.32 mm, and DV—.4 mm. Cannulas were fixed to the skull with anchoring screws and acrylic cement. After animals were euthanized, 30 μm cryogenic brain sections were made using a cryostat (Leica) and analyzed to determine cannula localization with histological staining.

### 2.4. Mechanical Allodynia Test

The mechanical withdrawal threshold was assessed using the up–down method with von Frey filaments as previously described [29]. Mice were acclimated to a wire‐mesh floor in Plexiglas cubicles for 1 h, after which mechanical sensitivity was evaluated using a set of calibrated von Frey fibers (0.008, 0.02, 0.04, 0.07, 0.16, 0.4, 0.6, 1.0, 1.6, and 2.0 g; Stoelting Co. Wood Dale, IL, USA) applied to the plantar surface of the hind paw for 5 s until the fibrils bent slightly. We considered the appearance of any of the following behaviors as a withdrawal response: (1) rapid flinch or withdrawal of the paw, (2) spreading of the toes, or (3) immediate licking of the paw. If the animal moved the paw for some other reason before the end of 2 s, the test was considered ambiguous and repeated. If a positive response was obtained to the first stimulus (i.e., the 0.16 g filament), then the next lower filament was applied; if there was no response, the next higher filament was used. When the first response appeared, the experiment was continued until four additional responses were obtained. The 50% paw withdrawal threshold value was then calculated.

### 2.5. Thermal Allodynia Test

Thermal allodynia was assessed with a Hargreaves apparatus as previously described [30]. The heat source (390‐Plantar Test, IITC Life Science) was placed with the guiding light pointing toward the plantar surface of the hind paw, and the thermal beam was turned on. Paw withdrawal would stop the test, and withdrawal latency was measured. Minimum and maximum cut‐offs were assigned at 1s and 20 s, respectively. Measurements were repeated five times in 5 min intervals on each paw, and the average of the five measurements was taken and analyzed.

### 2.6. Neuronal Stimulation

The 200 µm optic fibers in the cannulas were linked to the fiber optic rotary joint and then connected to a 473‐nm blue or 589‐nm yellow laser (NEWDOON, Hangzhou Newdoon Technology Co., Ltd.). The blue light stimulation parameters were 10 Hz, 10 nm wave width, and tip light intensity of 5 mw/mm^2^, whereas the yellow light stimulation was 10 Hz, wave width 50 nm, and tip light intensity 10 mw/mm^2^ [31]. Light stimuli were used simultaneously during the behavioral test; the test ended when the light was turned off. Then, there was a 5‐min break before the next test.

### 2.7. Administration of Drug

Specifically, DREADDs, such as human muscarinic acetylcholine receptor M3 designer receptor exclusively activated by designer drugs excitatory (hM3Dq) or human muscarinic acetylcholine receptor M4 designer receptor exclusively activated by designer drugs inhibitory (hM4Di), were used to activate or inhibit ACC neurons via CNO in a projection‐specific manner [32]. For chemical genetics, mice were injected intraperitoneally with 1 mg/kg of CNO for hM3Dq [33, 34], and 3 mg/kg of CNO for hM4Di [35]. EA stimulation was performed 15 min following injection.

### 2.8. EA Treatment

EA stimulation was performed 15 min following CNO injection. Stainless‐steel acupuncture needles (0.16 mm diameter, 13 mm length) were inserted bilaterally into the hind limb acupoints Zusanli (ST36) and Sanyinjiao (SP6). The needles were connected to a HANS acupoint nerve stimulator (HANS‐200 A, Huawei Co., Ltd., Beijing, China). Electrical stimulation was delivered at a frequency of 2 Hz, an intensity of 1.0 mA, using a continuous waveform for 30 min per session. Treatments were administered once daily for 7 days.

### 2.9. Western Blotting (WB)

Experimental mice were deeply anesthetized with 2%–3% sevoflurane and then ACCs were isolated. ACC neurons were collected and lysed in RIPA buffer containing protease inhibitor cocktails (Sigma–Aldrich). Then the protein samples were loaded on denatured sodium dodecyl sulfate gels for electrophoresis and subsequently transferred to nitrocellulose membranes and incubated with antibodies against mGluR5 (1:2000; Cell Signaling Technology, USA) or GAPDH (1:2500; Zenbio, China) as an internal control overnight at 4°C. The target bands were detected by HRP‐conjugated secondary antibodies and visualized with an AI800 ultra‐sensitive gel imager, quantified by Image J. Then the intensity of the mGluR5 band was normalized to the GAPDH intensity of the same sample.

### 2.10. Immunohistochemistry

Deeply anesthetized mice (3% sevoflurane) were perfused with 4% paraformaldehyde (PFA) in PBS. ACC was isolated and fixed in 4% PFA for 1.5 h and overnight. The tissue is embedded in paraffin, then sectioned at a thickness of 5 μm, floated on a 42 °C water bath, and baked in a 60 °C oven for 30 min. Xylene I for 5 min, Xylene II for 5 min, Xylene III for 5 min, anhydrous ethanol for 1 min, 95% ethanol for 1 min, 75% ethanol for 1 min, followed by washing in distilled water for 5 min. EDTA microwave heating for 5–8 min for antigen retrieval, then cooled to room temperature. Outline the area with an immunohistochemistry pen to prevent reagent overflow, add endogenous peroxidase blocking solution, and incubate at room temperature for 10 min, then wash with PBS buffer 3 times for 5 min each. Add blocking serum and incubate at 37 °C for 30 min, remove excess serum without washing. Add primary antibody c‐Fos (1:500, Abcam) and incubate in a 37°C humidified chamber for 2 h, wash with PBS buffer 3 times for 5 min each. Add HRP‐labeled antibody, incubate at 37 °C for 30 min, wash with PBS buffer 3 times for 5 min each. Prepare DAB chromogen solution by mixing 1 mL of B solution with 1 drop of A solution (Beyotime Biotechnology, China), add the DAB solution, and observe under the microscope (Leica, Thunder image 3D tissue, Germany).

### 2.11. Immunofluorescence (IF)

Mice were deeply anesthetized with isoflurane and then intracardially perfused with perfusion buffer, followed by 4% PFA in phosphate buffer (PB; pH 7.4); throughout, they were kept at room temperature (24–26°C) for 30 min. Brains were fixed in 4% PFA overnight at 4°C and then transferred to 30% sucrose in PBS to equilibrate for 3 days. Next, 10 μm coronal sections were cut on a cryostat (Leica) and washed with PBS for 10 min. Sections were then washed with PBS with 0.3% Triton X‐100 (PBST) for 10 min and incubated in PBST and 3% normal donkey serum (NDS) for 1 h at room temperature. Sections were then incubated with primary antibody (anti‐mGluR5: Cell Signaling Technology; anti‐c‐Fos: Abcam) overnight in PBST with 3% NDS at 4°C. Sections were then washed in PBS and incubated for 2 h at room temperature with Alexa Fluor 488 (1:500; Invitrogen) and Alexa Fluor 594 (1:500; Invitrogen) in 5% normal goat serum and NDS in PBST. Sections were washed in PBS and coverslipped with Vectashield mounting medium with DAPI. Slides were stored at 4°C until examined.

### 2.12. Proteomics

Collecting ACC region brain tissue for protein preparation for proteomics analysis. An amount of peptide sample corresponding to 2.5 μg of total protein was resolved in an Eksigent nanoLC ultra nanoflow high‐performance liquid chromatography in tandem with a TripleTOF6600+ mass spectrometer set for information‐dependent acquisition (IDA) and data‐independent acquisition (DIA) modes. The peptides were loaded onto a C18 column trap (Nano Trap RP‐1,3 μm 120 Å, 10 mm × 0.075 mm; Phenomenex, CA, USA) at a flow rate of 3 μL min^−1^ of 0.1% formic acid in water for 10 min to desalt and concentrate the sample, which was then resolved by HPLC using a stationary phase of a C18 analytical column (bioZen Peptide Polar C18 nanocolumn, 75 μm × 15 cm, particle size 3 μm, 120 Å; Phenomenex) with mobile phase gradients at a flow rate of 300 nL min^−1^ of 80% acetonitrile/0.1% formic acid for 60 min. The eluate was ionized and sprayed into the mass spectrometer using OptiFlow Turbo V Source (Sciex). Ion source gas 1, ion source gas 2, and curtain gas were set at 19, 0, and 25 vendor arbitrary units, respectively. The interface heater temperature was 150 °C and the ion spray voltage was 3.3 kV. Mass spectrometry was operated in the positive ion mode set for 3500 cycles per 105 min gradient elution. Each cycle performed one time of flight (TOF) scan (250 ms accumulation time, 350–1250 m/z window with a charge state of + 2) followed by IDA of the 100 most intense ions, while the minimum MS signal was set to 150 counts.

### 2.13. Statistical Analyses

Quantitative analysis of IF staining was performed using the Image J professional image analysis system. All statistical tests were performed with GraphPad Prism 9.0 software. Data are expressed as mean ± SEM. Sample sizes were determined based on previous studies in the field and our prior experience with similar experimental paradigms. The number of animals used in each experiment is indicated in the figure legends. Normality of the data distribution was assessed prior to applying parametric statistical tests. For comparisons between two groups were performed using a Student’s *t*‐test and comparisons among three groups were performed using ANOVA analysis. A Bonferroni post hoc test was used for further comparison. Statistical significance was set at *p*  < 0.05.

## 3. Results

### 3.1. Activation of ACC Excitatory Neurons Reduces Pain Threshold in Naïve Mice

Schematic of the experimental setup. Mice were fixed in a stereotaxic frame under prone positioning. Viral vectors and optical fibers were implanted into the ips ACC, defined as the hemisphere on the same side as the cranial surgery. A CCI was then induced in the con sciatic nerve, defined as the side opposite to the viral injection. In subsequent analyses, ips denotes manipulations or measurements on the injected hemisphere, whereas con refers to the con limb subjected to CCI (Figure 1A). Unilateral photoactivation of excitatory neurons in the ips ACC induced mechanical and thermal hypersensitivity in naïve mice. Specifically, the ChR2‐on group exhibited a significant reduction in paw withdrawal thresholds and a shortened latency to thermal stimulation compared with the ChR2‐off group (Figure 1B,C). These results were consistent with our previous research [36]. Elevated c‐Fos immunoreactivity confirmed robust neuronal activation within the ACC following blue light stimulation (Figure 1D). Conversely, yellow light‐mediated inhibition of ACC excitatory neurons increased both mechanical and thermal withdrawal thresholds in CCI mice (Figure 1E–G), indicating that suppression of pyramidal neuron activity in the ACC reversed hypersensitivity.

### 3.2. EA Reverses CCI‐Induced Hypersensitivity by Suppressing mGluR5

**Figure 2:**
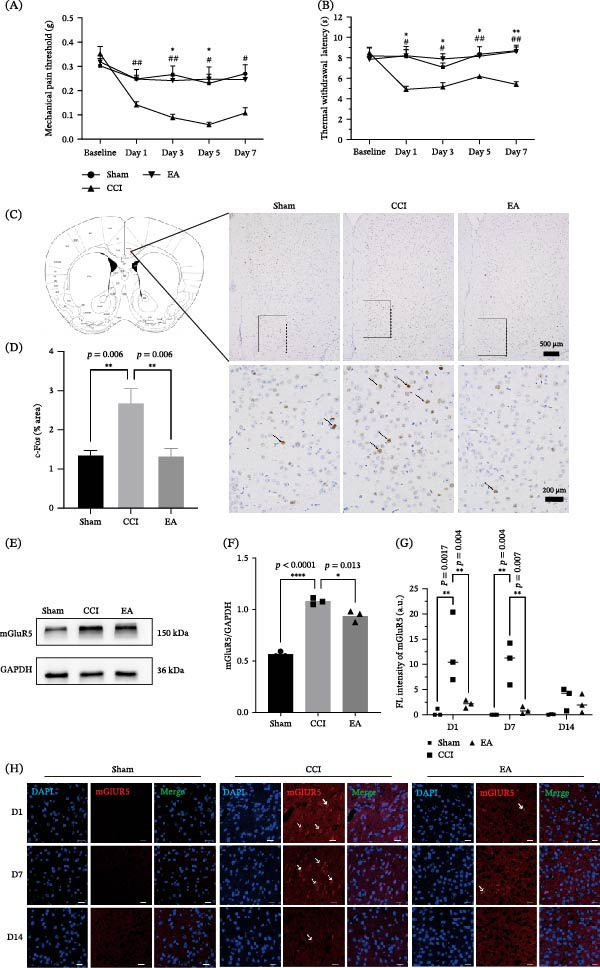
EA reverses CCI‐induced hypersensitivity and downregulates ACC mGluR5 expression. (A and B) EA treatment at ST36 and SP6 restored mechanical and thermal withdrawal thresholds in CCI mice (*n* = 7,  ^∗^ *p*  < 0.05, CCI group compared with sham group as assessed by repeated measures ANOVA with a Bonferroni post hoc test; ^#^ *p*  < 0.05, ^##^ *p* < 0.01, EA group compared with CCI group by two‐way ANOVA with Bonferroni post hoc test). (C and D) c‐Fos immunostaining in the ACC (ANOVA analysis,  ^∗∗^ *p* < 0.01). (E and F) Western blotting and statistical results (ANOVA analysis,  ^∗^ *p* < 0.05). (G and H) The mGluR5 of immunofluorescence and statistical results (bar = 20 µm) (ANOVA analysis,  ^∗∗^ *p* < 0.01). The white arrow represents mGluR5.  ^∗∗∗∗^ *p* < 0.0001.

In the CCI model, both mechanical and thermal withdrawal thresholds were significantly decreased, validating the successful induction of neuropathic pain. EA treatment at Zusanli (ST36) and Sanyinjiao (SP6) restored withdrawal thresholds, demonstrating a reversal of mechanical and thermal hypersensitivity (Figure 2A,B). c‐Fos immunostaining revealed that CCI markedly increased neuronal activation in the ACC, whereas EA treatment reduced c‐Fos expression to near‐sham levels (Figure 2C,D). Western blot and IF analyses further showed that CCI upregulated mGluR5 expression in the ACC across multiple time points, while EA treatment significantly downregulated mGluR5 levels (Figure 2E–H). We observed a decrease in mGluR5 expression in CCI mice on day 14, which is consistent with previous literature [37]. Collectively, these data demonstrate that EA attenuates hypersensitivity in CCI mice by inhibiting mGluR5 overexpression in the ACC.

### 3.3. Chemogenetic Activation of ACC Pyramidal Neurons Counteracts EA Analgesia

**Figure 3:**
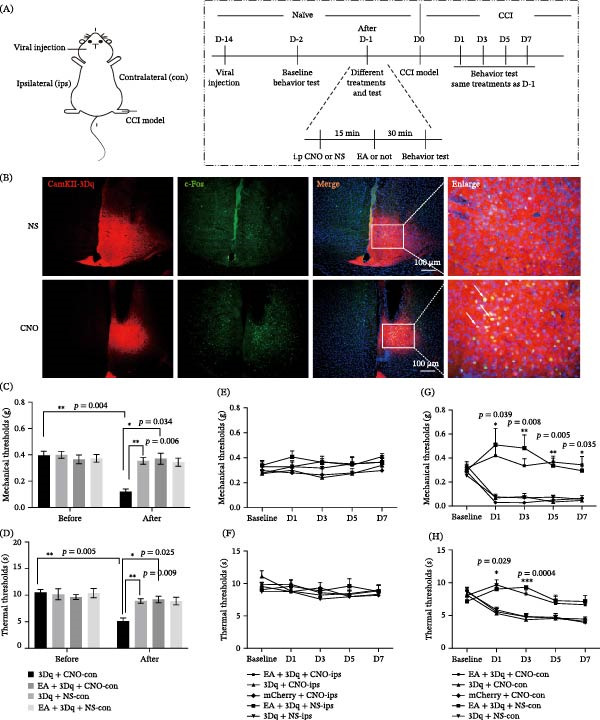
Chemogenetic activation of ACC pyramidal neurons counteracts EA analgesia. (A) Schematic illustration of the experimental process. (B) Intraperitoneal CNO (1 mg/kg) activated ACC neurons, as indicated by increased c‐Fos staining. (C and D) The mechanical and thermal thresholds of naïve mice before and after treatment (repeated measures ANOVA with Bonferroni post hoc test, *n* = 7,  ^∗^ *p* < 0.05,  ^∗∗^ *p* < 0.01). (E and F) The mechanical and thermal pain threshold of ipsilateral plantar threshold changes were observed on 1, 3, 5, and 7 days after CCI. (G and H) The mechanical and thermal pain threshold of contralateral plantar threshold changes were observed on 1, 3, 5, and 7 days after CCI (repeated measures ANOVA with Bonferroni post hoc test, *n* = 7,  ^∗^ as 3Dq + CNO group compared with the EA + 3Dq + CNO group,  ^∗^ *p* < 0.05, ^∗∗^ *p* < 0.01).  ^∗∗∗^ *p* < 0.001.

To assess whether ACC excitatory neurons contribute to the analgesic effect of EA, we selectively expressed hM3Dq in pyramidal neurons of the ips ACC. In naïve mice, intraperitoneal CNO administration (1 mg/kg) significantly activated these neurons, as verified by increased c‐Fos staining (Figure 3B). CNO‐induced activation of ACC excitatory neurons markedly decreased both mechanical and thermal thresholds compared with saline‐treated controls (Figure 3C,D), indicating that activation of ACC pyramidal neurons induces pain hypersensitivity in naïve mice. However, EA eliminates pain sensitization induced by chemogenetic CNO activation of ACC pyramidal neurons (Figure 3C,D), indicating that EA‐induced analgesic hyperalgesia may suppress excitatory neuronal activity in the ACC.

Next, we examined whether activation of ACC excitatory neurons affects EA‐induced analgesia in CCI mice. No changes were detected in ips withdrawal thresholds (Figure 3E,F). Activation of ACC excitatory neurons did not further reduce the pain threshold, suggesting that ACC excitatory neurons may be involved in neuropathic pain processes. However, when EA treatment was increased, EA analgesia remained significantly effective, indicating that EA may inhibit the activation of ACC excitatory neurons (Figure 3G,H). Moreover, thresholds did not differ between mCherry + CNO and hM3Dq + CNO groups (Figure 3G,H), further confirming that EA‐induced analgesia depends on the functional suppression of ACC excitatory neurons.

### 3.4. Chemogenetic Inhibition of ACC Pyramidal Neurons Mimics EA Analgesia

**Figure 4:**
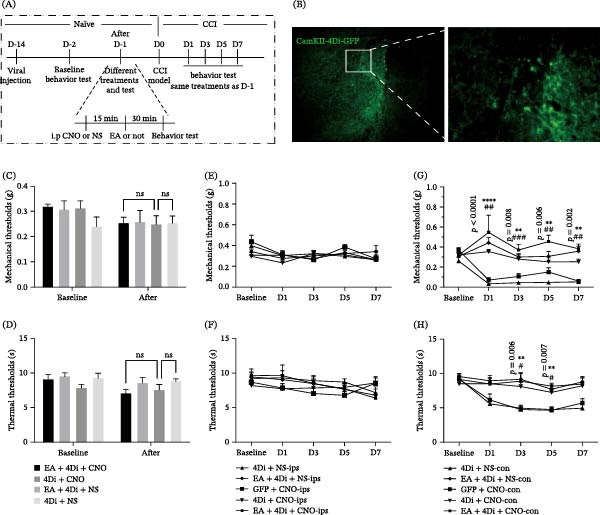
Chemogenetic inhibition of ACC pyramidal neurons mimics EA analgesia. (A) Schematic illustration of the experimental process. (B) AAV2/9‐CamKII‐hM4Di‐GFP expressed in the ACC. (C and D) The paw withdrawal thresholds and thermal pain threshold when inhibition of ACC excitatory neurons in naïve mice. (E and F) The mechanical and thermal pain threshold of ipsilateral plantar threshold changes were observed on 1, 3, 5, and 7 days after CCI. (G and H) The mechanical and thermal pain threshold of contralateral plantar threshold changes were observed on 1, 3, 5, and 7 days after CCI (repeated measures ANOVA with Bonferroni post hoc test, *n* = 8,  ^∗^ *p* < 0.05,  ^∗∗^ *p* < 0.01,  ^∗∗∗^ *p* < 0.001 as 4Di + NS‐con group and 4Di + CNO‐con (*p*‐value annotation); ^#^ *p* < 0.05, ^##^ *p* < 0.01, ^###^ *p* < 0.001 as EA + 4Di + NS‐con and 4Di + NS‐con group).  ^∗∗∗∗^ *p* < 0.0001.

Next, we examined whether suppression of ACC pyramidal neurons is sufficient to reproduce EA analgesia. Expression of hM4Di in the ACC followed by CNO administration inhibited excitatory neuronal activity (Figure 4B). In naïve mice, inhibition of ACC neurons did not alter baseline thresholds (Figure 4C,D). In contrast, in CCI mice, chemogenetic inhibition increased mechanical and thermal thresholds compared with saline controls (Figure 4G,H), thereby mimicking the analgesic effects of EA. Notably, the combination of EA and hM4Di activation (EA + 4Di + CNO) did not produce additive analgesia, consistent with the hypothesis that EA and chemogenetic inhibition act on a shared ACC‐dependent pathway. No ips changes were observed (Figure 4E,F) that EA achieves its analgesic effect by inhibiting pyramidal neurons in the ACC region.

### 3.5. Proteomic Alterations Associated With EA Intervention

**Figure 5:**
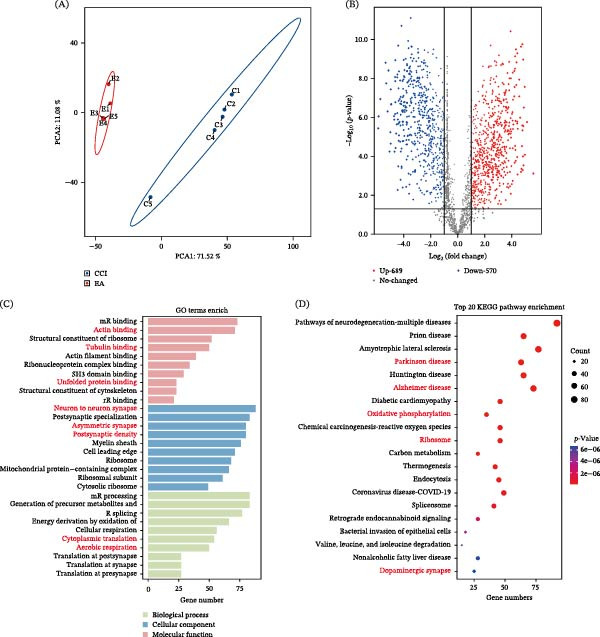
Proteomic alterations associated with EA intervention. (A) Principal component analysis showed clear separation between CCI and EA groups. (B) Volcano plot analysis identified 689 upregulated and 570 downregulated proteins after EA treatment. (C and D) GO and KEGG enrichment analysis.

The experimental results revealed significantly differentially expressed proteins between the experimental and control groups, along with their potential biological functions and pathways, through principal component analysis (PCA), GO functional enrichment analysis, and KEGG pathway enrichment analysis. PCA demonstrated distinct intergroup differences, with PC1 (71.52%) and PC2 (11.08%) collectively explaining the majority of data variation (cumulative > 80%) (Figure 5A), indicating significant disparities in protein expression profiles between the experimental and control groups. These differences may stem from alterations in neuronal activity (e.g., inhibition or activation of ACC pyramidal neurons) or neuroinflammatory/metabolic reprogramming. The volcano plot results show that after EA treatment, 689 proteins are upregulated and 570 proteins are downregulated (Figure 5B). The mGluR5 is a G protein‐coupled receptor abundantly expressed in the ACC, serves as a critical mediator of chronic pain through its regulatory roles in synaptic plasticity and neuronal excitability. Go‐enrichment functions analysis (Figure 5C) reveals its involvement in key biological processes, including postsynaptic density organization, where it interacts with NMDA receptors to modulate synaptic strength, and activity‐dependent protein synthesis at synapses via mTOR‐mediated translational control. KEGG‐enriched pathway analysis (Figure 5D) further associates mGluR5 with neurodegenerative disorders through mechanisms involving calcium‐mediated excitotoxicity, oxidative phosphorylation via mitochondrial dysfunction‐induced ROS production, and retrograde endocannabinoid signaling through 2‐AG‐dependent presynaptic inhibition of glutamate release. These data further support a role for EA in rebalancing excitatory signaling within the ACC through suppression of mGluR5‐mediated pathways.

## 4. Discussion

The viral constructs used in this study were driven by the CaMKIIα promoter, which predominantly labels excitatory pyramidal neurons in the cortex to manipulate glutamatergic principal neurons in cortical networks [38]. Notably, mGluR5 is highly expressed in postsynaptic compartments of excitatory neurons and plays an important role in regulating glutamatergic synaptic transmission [39, 40]. Therefore, the modulation of mGluR5 signaling may contribute to the observed changes in ACC pyramidal neuronal activity and the associated analgesic effects of EA. The present findings suggest a potential mechanistic link between EA‐induced behavioral analgesia and the modulation of ACC excitatory neuronal activity. In this study, neuronal activity recordings were primarily derived from neurons targeted by viral constructs driven by the CaMKIIα promoter, which preferentially labels cortical excitatory pyramidal neurons. Hyperexcitability of ACC pyramidal neurons has been widely reported in chronic pain states and is thought to contribute to enhanced pain perception and affective components of pain [41–43]. Consistent with this framework, our results show that EA treatment alleviated pain‐related behaviors while concurrently reducing mGluR5 activity in the ACC, suggesting that suppression of pyramidal neuron hyperactivity may underlie the observed behavioral improvements.

Importantly, our molecular analyses further revealed alterations in mGluR5 expression following EA treatment. mGluR5 is a postsynaptic metabotropic glutamate receptor that is highly expressed in cortical excitatory neurons and plays a crucial role in regulating glutamatergic synaptic transmission, intracellular Ca^2+^ signaling, and synaptic plasticity [44–46]. Previous studies have demonstrated that enhanced mGluR5 signaling contributes to cortical sensitization and persistent pain by promoting excitatory synaptic transmission within the ACC [12, 21]. Therefore, the reduction of mGluR5 expression observed in this study may represent a key molecular mechanism through which EA suppresses ACC pyramidal neuron excitability. Taken together, we support that EA alleviates neuropathic pain by modulating mGluR5‐dependent glutamatergic signaling in ACC excitatory neurons, thereby reducing cortical hyperactivity and ultimately improving pain‐related behavioral outcomes.

Using a CCI mouse model and a combination of optogenetics/chemogenetics, behavioral assays, and molecular readouts, we show that: (i) activating excitatory (pyramidal) neurons in the ACC reduced mechanical and thermal withdrawal thresholds (pronociceptive) in naïve mice, whereas inhibiting these neurons increased thresholds (antinociceptive); (ii) EA attenuated hypersensitivity and reduced ACC mGluR5 protein levels; and (iii) chemogenetic inhibition and EA did not produce additive analgesia, suggesting convergence on an ACC‐mGluR5‐dependent pathway. Together, these data link EA’s central analgesia to ACC pyramidal neuron excitability and mGluR5 signaling, adding mechanistic detail to the ACC’s role in integrating the sensory and affective dimensions of pain [9, 47].

The ACC’s bilateral connectivity and projections to descending control centers, such as the periaqueductal gray, may explain why unilateral manipulations influence bilateral thresholds and highlight the need for future studies addressing laterality and circuit specificity of EA [48–50]. Yi et al. [51] further showed in the formalin‐induced inflammatory pain model that con EA required an intact ACC for analgesia, whereas ips EA did not. This lateralized dependence aligns with our findings: ACC inhibition increased withdrawal thresholds, and the combination of ACC inhibition with EA showed no additivity. These results support the notion that con EA exerts its effects primarily via cortical circuits, where ACC suppression saturates analgesic capacity (“ceiling effect”), while ips EA may rely more on spinal/brainstem pathways. This framework explains why ACC manipulations can account for, but not fully cover, the analgesic spectrum of EA.

mGluR5‐dependent synaptic plasticity is closely associated with chronic pain. In cortical circuits, including the ACC, mGluR5 contributes to excitatory plasticity underlying hypersensitivity. Transient re‐expression of astrocytic mGluR5 during early chronic pain further enhances glutamatergic transmission, supporting an integrated neuron‐glia‐synapse pathology [15, 52]. Consistent with this framework, we observed that EA reduced CCI‐induced upregulation of ACC mGluR5. Previous studies report that EA suppresses mGluR5‐related signaling at both peripheral and central levels, attenuating hypersensitivity and inflammation [53, 54]. Our finding that EA reduced ACC mGluR5 expression, together with the lack of additivity between EA and chemogenetic inhibition, indicates convergence on the ACC‐mGluR5 axis. EA has also been shown to engage the endocannabinoid system (ECS), which suppresses glutamate release and promotes inhibitory synaptic plasticity in cortical circuits [55]. We therefore propose that EA dampens ACC excitability through coordinated modulation of glutamatergic, mGluR5, and ECS signaling.

Nierhaus et al. [56] demonstrated with EEG/fMRI that stimulation at acupoints elicits cortical responses distinct from nonacupoint stimulation, indicating selective modulation of pain‐related networks. This indicates that acupoint stimulation is not simply equivalent to tactile input but can selectively modulate pain‐related network nodes. Our findings replicate and refine this observation at the ACC: in CCI mice, EA reduced ACC mGluR5 expression and increased withdrawal thresholds, while optogenetic/chemogenetic inhibition of ACC excitatory neurons produced an effect nonadditive with EA. Together, these results highlight the ACC‐mGluR5 axis as a critical convergence point. Thus, the framework of “acupoint‐specific responses converging on ACC” provides a concrete molecular and circuit‐level anchor for acupuncture neuroscience.

The combination of EA and chemogenetics did not exhibit an additive effect on pain behavior induced by inhibition of ACC pyramidal neurons, which is compatible with shared pathway/downstream effect saturation: once ACC firing is suppressed or mGluR5 is reduced, the remaining room for additional EA‐driven modulation is limited. From a systems perspective, this may reflect a ceiling effect within ACC‐descending inhibitory circuits. This interpretation is coherent with the circuit and molecular evidence above [9, 52].

Our proteomics implicate postsynaptic density organization, mTOR‐related translation, mitochondrial function, and ECS‐related signaling, which are processes tied to excitotoxicity, metabolic stress, and plasticity remodeling in chronic pain. Integrating these findings with prior ACC electrophysiology and cell biology, we propose a working model: CCI drives glutamatergic overactivation and mGluR5‐dependent plasticity in the ACC; EA reduces mGluR5 and upstream glutamatergic drive and may recruit ECS‐mediated synaptic inhibition to reverse/attenuate pathological excitability and plasticity [52].

It should be noted that the cortical microcircuits of the ACC involve complex interactions between excitatory pyramidal neurons and various inhibitory interneurons. Although our recordings primarily reflect activity changes in CaMKIIα‐expressing pyramidal neurons, the excitability of these neurons is regulated by local inhibitory networks (including interneurons expressing microfibrillar protein and somatostatin). Therefore, the observed reduction in pyramidal neuronal activity following EA therapy may not directly affect the pyramidal neurons themselves but could also result from modulation by upstream inhibitory neural circuits. The cellular heterogeneity of ACC (including inhibitory interneurons, astrocytes, and microglia) and its long‐range projections (such as the ACC‐PAG pathway and cortical–cortical pathways) require further in‐depth investigation. Future studies should incorporate cell type‐specific manipulation, circuit‐specific tracing, and closed‐loop intervention strategies to elucidate the precise microcircuit mechanisms underlying the analgesic effects of EA. Additionally, several limitations of this study warrant clarification: first, the results were based on a single neuropathic pain model (CCI), and future validation using more neuropathic or inflammatory pain models will enhance the generalizability of the findings; second, the study was conducted exclusively in animal models, and clinical studies are needed to confirm the existence of similar mechanisms in humans; third, clinical EA protocols exhibit significant variations in stimulation parameters, acupoint selection, and treatment duration, which may influence therapeutic outcomes; finally, this study did not directly validate the causal role of mGluR5 pharmacological intervention in early activation‐induced analgesia.

In conclusion, EA alleviates neuropathic pain possibly through modulation of mGluR5‐associated signaling pathways that regulate ACC pyramidal neuronal activity. Bidirectional manipulations demonstrated that activation of ACC pyramidal neurons reduced withdrawal thresholds (pronociceptive), whereas their inhibition increased thresholds (antinociceptive). Chemogenetic inhibition and EA did not produce additive effects, indicating convergence on an ACC‐mGluR5‐dependent pathway. These findings support that EA suppresses ACC pyramidal excitability, at least in part, by inhibiting mGluR5 signaling.

## Author Contributions

All authors made a significant contribution to the work reported, whether that was in the conception, study design, execution, acquisition of data, analysis, and interpretation, or all these areas, and took part in drafting, revising, or critically reviewing the article.

## Funding

This research was funded by the National Natural Science Foundation of China (Grants 82104622, 82575198, and 82501477), the Wenzhou Science and Technology Bureau (Grant Y20210787), and the Zhejiang Natural Science Foundation (Grant LQ24H310013).

## Disclosure

All authors gave the final approval of the version to be published, have agreed on the journal to which the article has been submitted, and agree to be accountable for all aspects of the work.

## Ethics Statement

The animal study protocol was approved by Laboratory Animal Ethics Committee of First Affiliated Hospital of WenZhou Medical University Institutional Animal Care and Use Committee (Number WYYY‐AEC‐2023‐010) for studies involving animals.

## Conflicts of Interest

The authors declare no conflicts of interest.

## Data Availability

The processed proteomics data supporting the findings of this study have been deposited in Figshare under the DOI: 10.6084/m9.figshare.30305986. The dataset includes normalized protein quantification values, differential expression results between the CCI and EA groups.
